# Simultaneous Tricuspid and Pulmonic Valve Replacement Due to Infective Endocarditis

**DOI:** 10.7759/cureus.31902

**Published:** 2022-11-26

**Authors:** Erol Belli, Luis Paulino, Mohamed Khayata, Nidhi Patel

**Affiliations:** 1 Department of Cardiothoracic Surgery, University of South Florida Morsani College of Medicine, Tampa, USA; 2 Department of Medicine, University of South Florida Morsani College of Medicine, Tampa, USA; 3 Department of Cardiovascular Sciences, University of South Florida Morsani College of Medicine and Tampa General Hospital Heart and Vascular Institute, Tampa, USA

**Keywords:** valve replacement, intravenous drug use, pulmonic valve, tricuspid valve, infectious endocarditis

## Abstract

Right-sided valvular infective endocarditis (RSIE) is often associated with intravenous (IV) drug abuse and typically involves the tricuspid valve. The involvement of both the tricuspid and pulmonic valves is a rare entity. A 39-year-old woman presented with fever, dyspnea on exertion, and chest pain. She was subsequently found to have infectious endocarditis (IE) with the involvement of both the tricuspid and pulmonic valves. Simultaneous tricuspid and pulmonic valvular repair with bioprosthetic valves were performed with bovine pericardium to reconstruct the anterior surface of the pulmonary artery. Recovery was complicated by the development of a complete atrioventricular (AV) block requiring pacemaker implantation. Following device placement, the patient also developed two episodes of ventricular tachycardia arrest likely precipitated by the device. Return of spontaneous circulation (ROSC) was achieved and no further episodes occurred once the device was exchanged with a cardiac resynchronization therapy defibrillator. The patient improved clinically and was discharged home with no further complications.

## Introduction

Right-sided infectious endocarditis (RSIE) is less common than left-sided infectious endocarditis (IE), accounting for only 5-10% of all IE cases [1]. However, the prevalence of RSIE has increased in the United States over the last 20 years and is likely explained by an increase in the use of central venous catheters, intracardiac devices, and intravenous (IV) drug abuse. Among RSIE cases in the United States, about 90% involve only the tricuspid valve. Isolated pulmonic valve endocarditis (PVE) is extremely rare, accounting for less than 2% of IE cases [1]. We present an exceptionally rare case of a patient with bi-valvular endocarditis involving both the tricuspid and pulmonic valves and describe the technical details of simultaneous tricuspid and pulmonic valve replacement.

## Case presentation

History of presentation

A 39-year-old female presented to the emergency department with a fever, chest pain, dyspnea on exertion, and bilateral lower extremity swelling. The patient’s symptoms had gradually worsened over the prior three weeks. She denied having any dizziness, syncope, nausea, vomiting, or palpitations. Vitals signs on presentation showed a temperature of 97.7 °F, heart rate of 127 beats/minute, respiratory rate of 27 breaths per minute, blood pressure of 138/96 mm Hg, and oxygen saturation of 96% on room air. Physical examination revealed a 2/6 systolic murmur throughout the pericardium and 2+ pitting edema in the lower extremities. Lungs were clear to auscultation bilaterally. No carotid bruits were appreciated, and jugular venous pressure was normal. Needle track marks were noted on skin examination in the upper extremities bilaterally.

Past medical history 

The patient reported a history of opioid abuse for the past 10-15 years. She has tried buprenorphine-naloxone therapy multiple times with some success, achieving intermittent sobriety for months to years at a time. She recently relapsed and started using IV heroin after the death of a close family member, and last used it one day prior to the presentation.

Differential diagnosis

Differential diagnosis includes infective endocarditis, acute decompensated heart failure, valvular dysfunction, and a perivalvular abscess. 

Investigations

**Figure 1:**
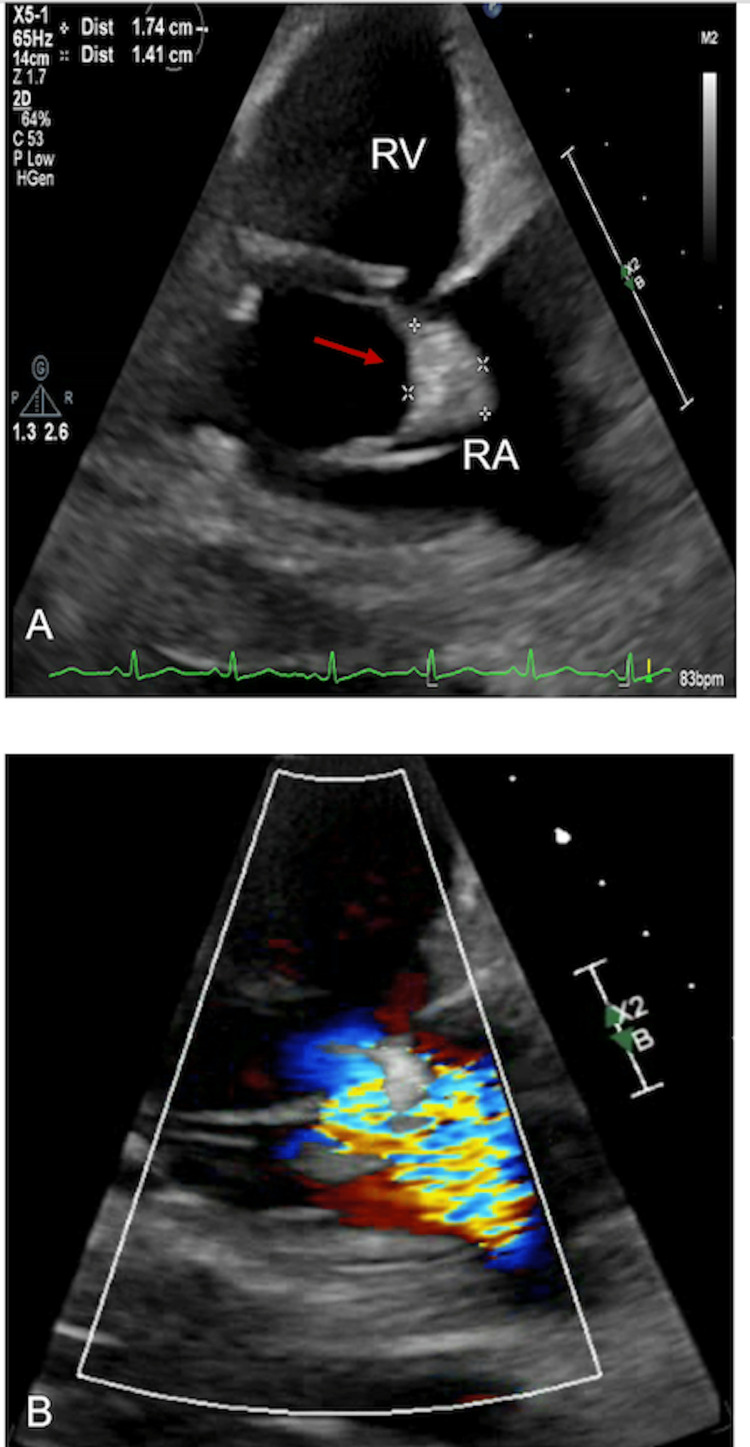
(A) 14 mm × 17 mm vegetation on the anterior leaflet of the tricuspid valve identified by the red arrow (arrow). (B) Color Doppler showing a large regurgitative jet. RV: right ventricle; RA: right atrium.

**Figure 2:**
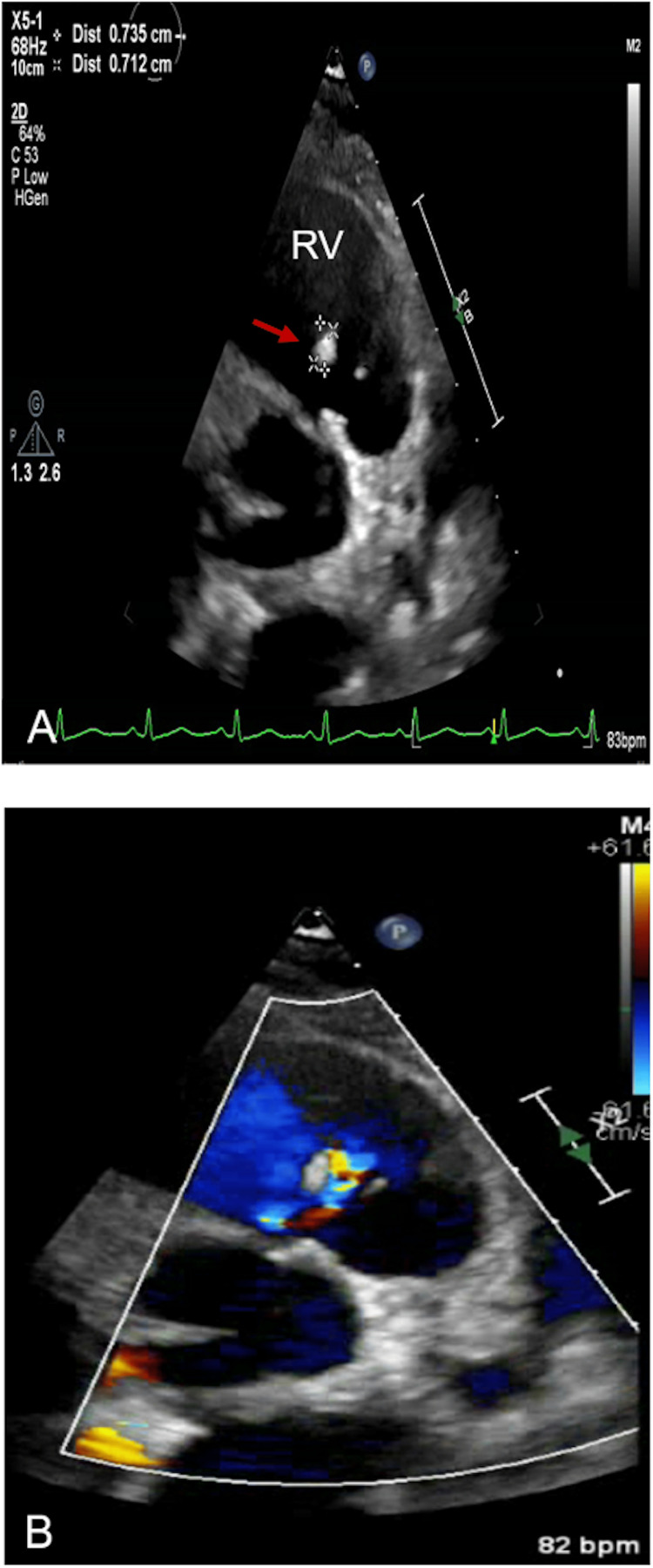
(A) 7 mm × 3 mm vegetation on the pulmonic valve (arrow). (B) Color Doppler of the pulmonic valve. RV: right ventricle.

**Figure 3:**
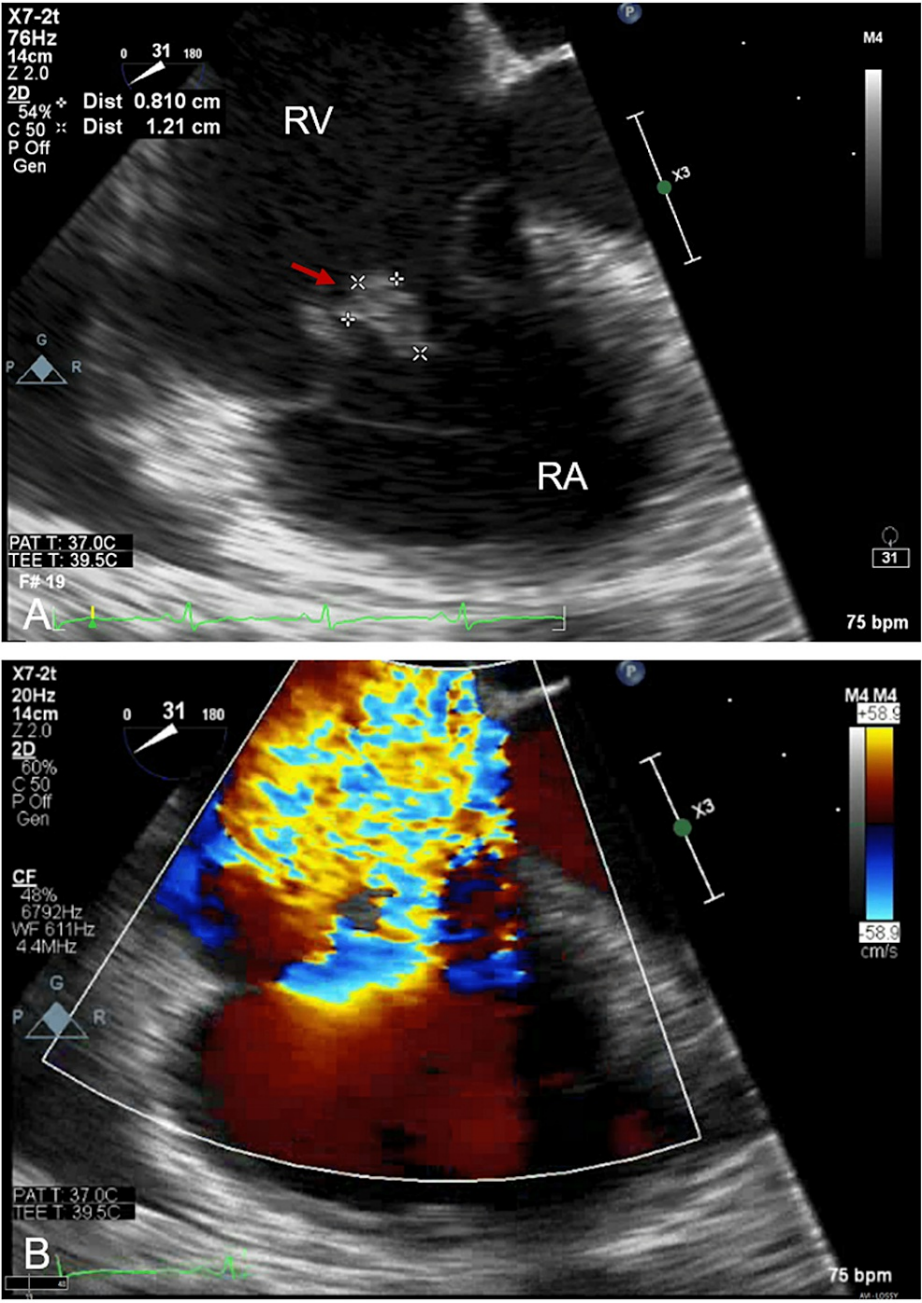
(A) Preoperative TEE demonstrating a vegetation on the anterior leaflet of the tricuspid valve (arrow). (B) Preoperative TEE with color Doppler showing a flail anterior leaflet of the tricuspid valve with severe tricuspid regurgitation. RV: right ventricle; RA: right atrium; TEE: transesophageal echocardiogram.

**Figure 4:**
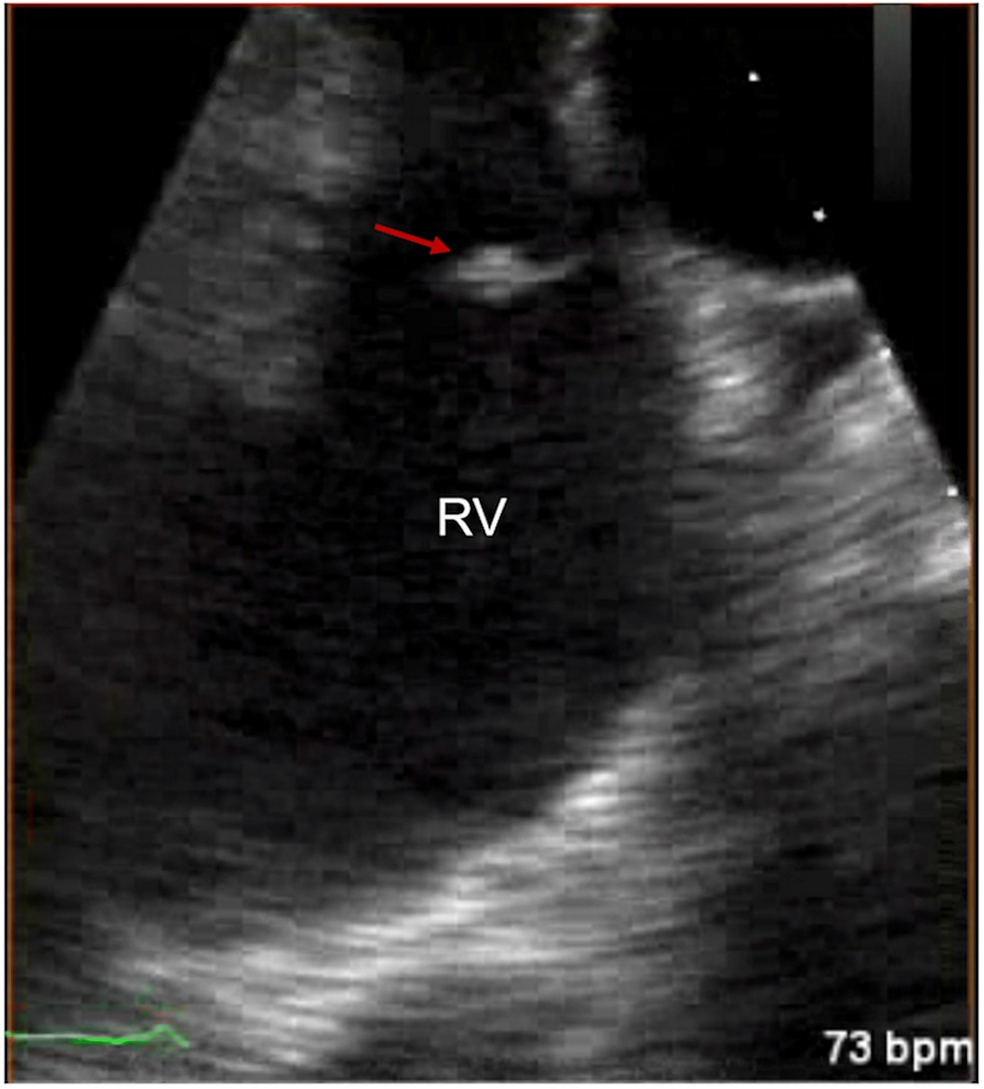
Preoperative TEE demonstrating a 7 mm × 3 mm pulmonic valve vegetation (arrow). RV: right ventricle.

Initial blood cultures obtained at presentation grew methicillin-susceptible Staphylococcus aureus (MSSA). A computed tomography (CT) angiography demonstrated numerous cavitary pulmonary lesions bilaterally. Transthoracic echocardiography (TTE) showed echo densities suspicious for vegetation on the tricuspid with moderate to severe tricuspid regurgitation noted on color Doppler (Figure 1(A)-1(B)). The pulmonic valve was also found to have vegetation with some evidence of regurgitation seen on the color Doppler (Figure 2(A)-2(B)). This study was followed up with a transesophageal echocardiogram (TEE), which demonstrated a flail anterior leaflet on the tricuspid valve with the presence of vegetation measuring 14 mm × 17 mm and severe tricuspid regurgitation (Figure 3). A mobile vegetation (7 mm × 3 mm) was also noted on the pulmonic valve (Figure 4). Left heart catheterization and coronary angiography showed patent coronary arteries.

Management

The patient was started on IV antibiotics for MSSA bacteremia. The cardiothoracic surgical team was consulted for evaluation of surgical candidacy for bi-valvular valve replacement and a decision was made to move forward with the surgical procedure.

**Figure 5:**
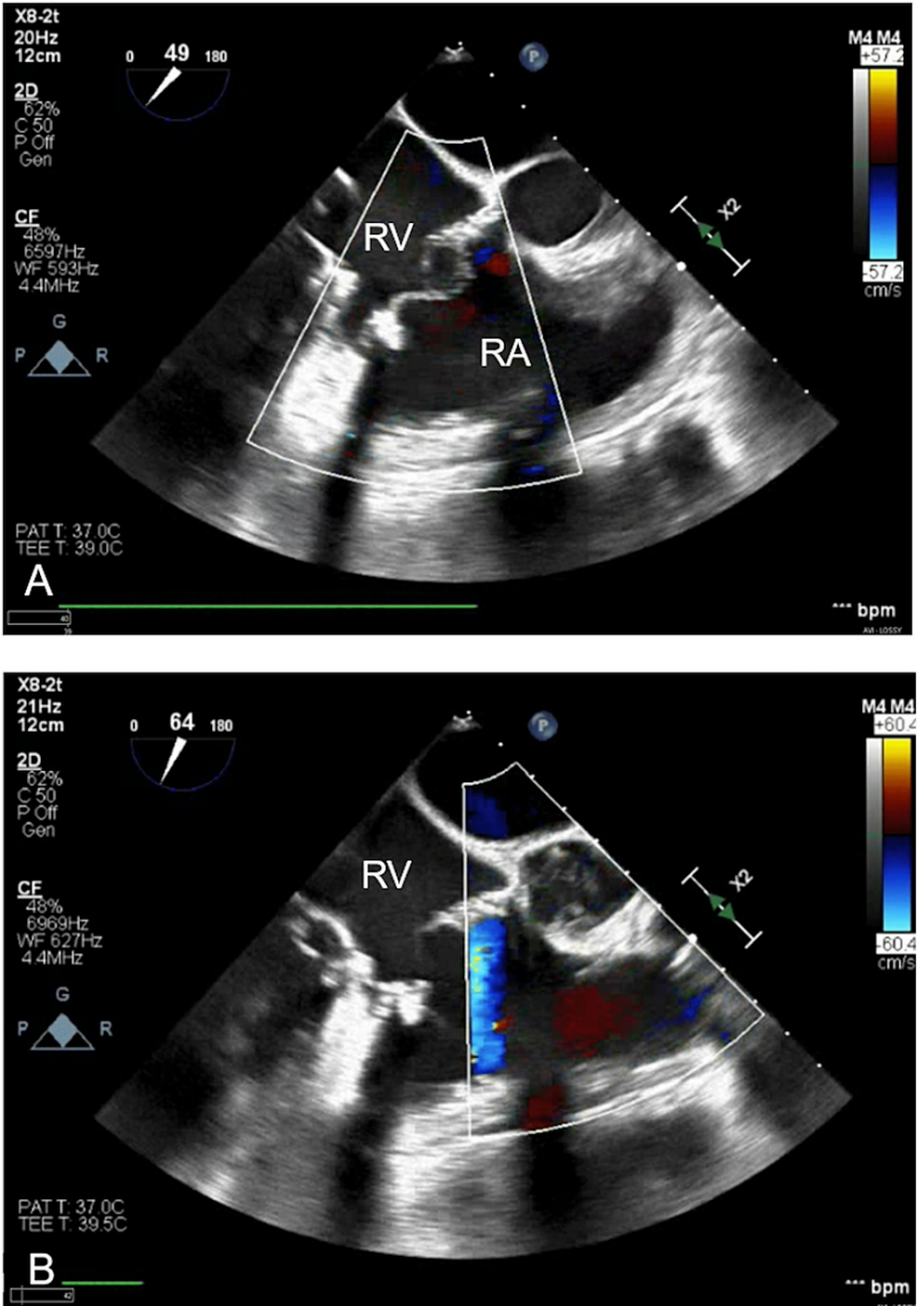
(A) Intraoperative TEE with color Doppler showing the bioprosthetic tricuspid valve. (B) Intraoperative TEE with color Doppler showing the bioprosthetic pulmonic valve. RV: right ventricle; RA: right atrium.

Following general endotracheal anesthesia, the patient was placed on cardiopulmonary bypass followed by aortic cross-clamp placement. Diastolic cardiac arrest was achieved with antegrade Del Nido cardioplegia. The tricuspid valve was exposed directly through the right atrium. The tricuspid valve inspection revealed an anterior leaflet perforation with vegetation on the anterior and septal leaflets. The leaflets were excised and debrided to healthy tissue. Circumferential 2-0 ethibond sutures were placed equidistant around the annulus and then around a 33 mm bioprosthetic Epic™ valve. The valve was tied down in place, followed by a saline float test, which showed excellent coaptation. The pulmonary valve was exposed directly through the pulmonary artery and carried across the pulmonary valve annulus. The leaflets were excised and debrided to healthy tissue. 4-0 prolene sutures were placed equidistant around the annulus and around a 29 mm bioprosthetic Epic™ valve for two-thirds of the distance around the valve. A piece of the bovine pericardium was then used to reconstruct the anterior surface of the pulmonary artery until reaching the valve. The bioprosthetic valve was then allowed to have a small tilt towards the branch of the pulmonary arteries and had the last third of the valve cuff sewn to the patch. The valve was then tied down in place, followed by a saline float test, which showed excellent coaptation. The patch was then brought transannular, enlarging the right ventricular outflow tract with 4-0 prolene. An intraoperative TEE was used to visualize the replaced valves for evaluation of placement and regurgitation, which did not demonstrate a regurgitative jet from either bioprosthetic valve (Figure 5). The total cardiopulmonary bypass time was 106 minutes.

The patient was extubated and successfully weaned off pressors and inotropes. However, she was found to have a complete atrioventricular (AV) block with a junctional escape rhythm on post-operative (PO) day 1, requiring external pacing. Given the persistence of the AV block, the decision was made to place a Micra™ pacemaker on PO day 5 as a treatment for her bradycardia. Later that same evening, the patient went into polymorphic ventricular tachycardic (VT) arrest, requiring chest compressions and direct current cardioversion (DCCV) to achieve a return of spontaneous circulation (ROSC). The patient was intubated and subsequently extubated the following day. On PO day 7, the patient went into VT arrest and ROSC was achieved once again. The patient was then evaluated by the electrophysiology team, who suspected the Micra™ pacer could be causing dysrhythmias in the setting of a right ventricular outflow tract bovine patch. The decision was made to remove the Micra™ pacemaker and place a cardiac resynchronization therapy defibrillator to allow for bi-ventricular pacing. The patient recovered clinically and was eventually discharged to home.

## Discussion

The diagnosis of IE is based on the Duke criteria, which utilizes clinical, laboratory, and echocardiography findings to determine the likelihood of the diagnosis. This patient met two of the major clinical criteria, having positive blood cultures and evidence of endocardial involvement on echocardiography, confirming a diagnosis of IE.

The primary imaging technique used in detecting RSIE is echocardiography. A TTE is especially useful when evaluating the right side of the heart given that right-sided cardiac structures are anatomically located anteriorly and therefore closer to the transducer. Further imaging with a TEE is recommended in patients with high-risk clinical or echocardiographic features such as new AV block, persistent fevers despite appropriate antibiotic coverage, Staphylococcus bacteremia, large or mobile vegetation, or valvular insufficiency. A CT scan can also be useful in patients with RSIE to evaluate for metastatic infections given that some of the most common complications include abscess formation and septic pulmonary embolism.

Management of tricuspid endocarditis involves the use of IV antibiotics. Indications for surgical intervention are based on the severity of the diagnosis and include persistent bacteremia despite adequate antimicrobial coverage, recurrent pulmonary emboli, abscess formation, right heart failure secondary to tricuspid regurgitation, or large, persistent tricuspid valve vegetations [2]. The use of prosthetic materials should be avoided when possible. Current guidelines do not specifically address the management of RSIE involving both the pulmonic and tricuspid valves, the patient met multiple indications for surgical intervention based on guidelines for single-valve IE. We present an exceptionally rare case of a patient with bi-valvular RSIE who successfully underwent simultaneous tricuspid and pulmonic valve replacement using the technique described above. This case demonstrates that this procedure can be performed in one surgical operation successfully and may be the preferred technique for patients requiring multi-valvular surgical repair for patients with IE.

A systematic review and meta-analysis of a cohort of 646 patients documented the most common complications and post-operative mortality of patients undergoing tricuspid valve replacement [3]. The most common complication was found to be prolonged ventilation, followed by recurrent endocarditis. The need for reoperation occurred in 14% of patients, while the 30-day post-operative mortality was found to be 7%. Unfortunately, there is limited data published on the outcomes of patients who have undergone pulmonary valve replacement due to IE, given that the tricuspid valve is much more commonly involved.

Another common complication of cardiac valvular surgery is the development of electrical abnormalities, including complete atrioventricular (AV) block, which developed in this patient following the procedure. Complete AV block typically occurs within 48 hours of surgery and has been noted to be the strongest predictor for pacemaker dependence [4]. The need for permanent pacemaker (PPM) placement has been previously demonstrated to be higher after undergoing valvular surgery involving the tricuspid valve when compared to other cardiac valves [5]. This is likely explained by the proximity of the tricuspid valve to the AV node, which may often undergo surgical injury or become disrupted due to cardiac tissue edema. One study reported the need for PPM placement in 21-22% of patients who underwent cardiac surgery involving the tricuspid valve [6]. No definitive guidelines have been published for when a permanent pacemaker (PPM) should be implanted, but current literature generally recommends PPM placement no earlier than 5-7 days from the date of surgery. A decision was made to place a Micra™ pacemaker on post-operative day 5 to address the patient’s complete AV block.

The patient’s hospital course was further complicated by two episodes of polymorphic ventricular tachycardia (VT) arrest. Although the development of VT arrest is an uncommon complication associated with cardiac valvular surgery, the patient did undergo a Micra™ pacemaker placement, which has previously been associated with episodes of ventricular tachycardia and cardiac arrest [7]. The device was exchanged for a cardiac resynchronization therapy defibrillator with no further episodes of polymorphic VT arrest reported. The patient recovered clinically and was medically cleared for discharge.

## Conclusions

In summary, bi-valvular endocarditis involving the tricuspid and pulmonic valves is a rare phenomenon with minimal published information on the management of this condition. The use of echocardiography and CT imaging serve as useful tools in the diagnosis and management of RSIE. Multidisciplinary care involving cardiologists, infectious disease specialists, and cardiothoracic surgeons was integral in providing a successful outcome for the patient. This case highlights the importance of multimodality imaging, addresses complications associated with cardiac valvular surgery, and provides a surgical approach for simultaneous tricuspid and pulmonic bi-valvular replacement.

## References

[1] Shmueli H, Thomas F, Flint N, Setia G, Janjic A, Siegel RJ (2020). Right-sided infective endocarditis 2020: challenges and updates in diagnosis and treatment. J Am Heart Assoc.

[2] Habib G, Lancellotti P, Antunes MJ (2015). 2015 ESC Guidelines for the management of infective endocarditis: The Task Force for the Management of Infective Endocarditis of the European Society of Cardiology (ESC). Endorsed by: European Association for Cardio-Thoracic Surgery (EACTS), the European Association of Nuclear Medicine (EANM). Eur Heart J.

[3] Luc JG, Choi JH, Kodia K (2019). Valvectomy versus replacement for the surgical treatment of infective tricuspid valve endocarditis: a systematic review and meta-analysis. Ann Cardiothorac Surg.

[4] Kim M, Deeb G, Eagle K (2001). Complete atrioventricular block after valvular heart surgery and the timing of pacemaker implantation. Am J Cardiol.

[5] Mar PL, Angus CR, Kabra R (2017). Perioperative predictors of permanent pacing and long-term dependence following tricuspid valve surgery: a multicentre analysis. Europace.

[6] Sunjic I, Shin D, Sunjic KM (2020). Incidence of atrioventricular block after valve replacement in carcinoid heart disease. Cardiol Res.

[7] Olsen FJ, Højlund S, Jacobsen MD (2018). Malignant ventricular tachycardia and cardiac arrest induced by a Micra™ leadless pacemaker. J Electrocardiol.

